# Sustained productivity in recombinant Chinese Hamster Ovary (CHO) cell lines: proteome analysis of the molecular basis for a process-related phenotype

**DOI:** 10.1186/1472-6750-11-78

**Published:** 2011-07-24

**Authors:** Paula Meleady, Padraig Doolan, Michael Henry, Niall Barron, Joanne Keenan, Finbar O'Sullivan, Colin Clarke, Patrick Gammell, Mark W Melville, Mark Leonard, Martin Clynes

**Affiliations:** 1 National Institute for Cellular Biotechnology, Dublin City University, Dublin 9, Ireland; 2 Bio-Manufacturing Sciences group, Pfizer, Inc., Grange Castle International Business Park, Clondalkin, Dublin 22, Ireland; 3 Bioprocess R&D, Pfizer Inc., 1 Burtt Rd, Andover, MA 01810, USA

## Abstract

**Background:**

The ability of mammalian cell lines to sustain cell specific productivity (Qp) over the full duration of bioprocess culture is a highly desirable phenotype, but the molecular basis for sustainable productivity has not been previously investigated in detail. In order to identify proteins that may be associated with a sustained productivity phenotype, we have conducted a proteomic profiling analysis of two matched pairs of monoclonal antibody-producing Chinese hamster ovary (CHO) cell lines that differ in their ability to sustain productivity over a 10 day fed-batch culture.

**Results:**

Proteomic profiling of inherent differences between the two sets of comparators using 2D-DIGE (Difference Gel Electrophoresis) and LC-MS/MS resulted in the identification of 89 distinct differentially expressed proteins. Overlap comparisons between the two sets of cell line pairs identified 12 proteins (AKRIB8, ANXA1, ANXA4, EIF3I, G6PD, HSPA8, HSP90B1, HSPD1, NUDC, PGAM1, RUVBL1 and CNN3) that were differentially expressed in the same direction.

**Conclusion:**

These proteins may have an important role in sustaining high productivity of recombinant protein over the duration of a fed-batch bioprocess culture. It is possible that many of these proteins could be useful for future approaches to successfully manipulate or engineer CHO cells in order to sustain productivity of recombinant protein.

## Background

Chinese hamster ovary (CHO) cells are the most widely used vehicle for the production of biopharmaceuticals due to their high productivity, robust nature, track record in industry, and their safety record [1]. There has been considerable success in developing high-producing CHO cell culture processes using approaches such as optimisation of media formulation, improvements in expression vector design and also improvements in the design of bioreactors [2,3]. However, the bottlenecks in the cellular machinery for the efficient production of recombinant proteins are poorly understood. If there are to be considerable improvements in increasing cellular productivity, a fundamental understanding of the biology underpinning productivity of these cells is required.

There have been a number of studies published using expression microarray and proteomic technologies to gain insights into the biology of mammalian cell lines used for biopharmaceutical production (reviewed [4,5]). Some studies have directly compared cell lines producing different levels of recombinant protein (from low to high producers) at one point in time, usually during the mid-exponential phase of growth [6-10]. Other studies have used profiling tools to deduce why media supplements such as butyrate [11-13] and DMSO [14], environmental conditions such as hyperosmotic pressure [15], or temperature shift [16], result in an increase in productivity. These studies have revealed many genes and proteins that are altered under such conditions and are related to diverse biological functions such as protein folding and secretion, cell metabolism, cytoskeletal architecture, cell growth or apoptosis. However, very few of these studies have investigated the molecular mechanisms that enable a recombinant cell line to sustain high levels of cell specific productivity over the full duration of a culture period, although Stansfield et al. [17] found that while large changes in monoclonal antibody cell specific productivity (qMAB) occur during a fed-batch culture of GS-NS0 cells (up to 6-fold), the cellular proteome remained remarkably constant, varying primarily with cell growth rate.

In this study, we present a proteomic analysis focussing specifically on a sustained productivity phenotype over the entire production culture. Comparative analysis was carried out on two CHO production cell line pairs, with each pairing differing markedly in their ability to sustain high productivity of MAB over a ten day culture period. All four clonal cell lines used in the study initially demonstrated high cell specific productivities; however, two out of the four cell lines were unable to sustain Qp over the full 10 days of fed-batch culture, despite an optimised culture process being employed. The aim of this study was to identify key proteins that may be associated with the ability of CHO cells to sustain Qp in suspension culture over a 10 day fed-batch culture using 2D-DIGE (Difference Gel Electrophoresis) and LC-MS/MS.

## Methods

### Cell culture and sample collection

Four CHO MAB-secreting cell lines were used in the study; two cell lines which were able to sustain productivity (clone 3B12 and clone 2.8) and two cell lines which were unable to sustain productivity over 10 days in culture (clone 5B5 and clone 1.14). All four cell lines were derived from the same host lineage and bank (CHO K1). Clones 2.8 and 1.14 express the same monoclonal antibody and were derived from the same transfection event. Clones 3B12 and 5B5 each express different monoclonal antibodies. All four cell line samples were derived from 2L bioreactor fed-batch cultures and were grown in identical proprietary serum-free media in suspension culture at 37°C followed by a temperature shift to 31°C after the initial exponential growth phase. All cell lines were seeded at a target seed density of 0.30 × 10^6 ^cells/mL and cell counts from bioreactors were measured using a Cedex Automated Cell Culture Analyzer (Roche Innovatis). Samples for 2D-DIGE analysis were collected at Day 3 (pre-temperature shift) during the exponential growth phase, at Day 7 (stationary phase) and at Day 10 (late stationary/death phase). Three separate bioreactor runs were set up for each of the four cell lines. This allowed us to collect three biological replicate samples at each of the three time points for each of the four cell lines. The cell pellets containing 2 × 10^7 ^viable cells were washed twice with cold phosphate-buffered saline (PBS-A) solution and then stored at -80°C.

### Determination of cell-specific productivity (Qp)

The concentration of protein product in conditioned media samples (volumetric titre) was determined by Protein-A HPLC. Cell specific productivity (pg protein/cell/day) (Qp) was determined by the following equation as recently published by our group [18].

where,

### 2D Difference Gel Electrophoresis (2D-DIGE)

Frozen cell pellets containing 2 × 10^7 ^cells were thawed and resuspended in 500: L of lysis buffer (7 M urea, 2 M thiourea, 30 mM Tris, 4% CHAPS, 5 mM magnesium acetate, pH 8.5), and then homogenised by carefully passing the samples through a 20 gauge needle 5 times. Samples were left on a shaker for 1 hr at room temp to allow extraction to take place, and then centrifuged at approximately 14,000 rpm (or equivalent g) for 15 min at 10°C to remove insoluble material. The supernatant was removed and stored at -80°C until required for use. Protein concentrations were determined using the thiourea-compatible Bradford protein assay (Bio-Rad) and found to be similar among all samples. As a result, 50 μg of protein was used per sample on each 2D-DIGE gel for comparative proteomic analysis experiments. Immobilized 18 cm linear pH gradient (IPG) strips (GE Healthcare), pH 4-7, were chosen for 2D-DIGE analysis as these gave good resolution of protein spots in a relatively broad pI range using the total cellular lysate protein extraction procedure described above. Previous optimisation experiments in our laboratory using 3-10 and 3-11NL IPG strips showed streaking in the basic pI range, hence the decision to use the pI 4-7 strips. However, it is recognised that any particular pH gradient and protein extraction procedure will resolve a limited number of proteins.

IPG strips were rehydrated in rehydration buffer (7 M urea, 2 M Thiourea, 4% CHAPS, 0.5% IPG Buffer, 50 mM DTT) overnight. Three biological replicate samples from each time point were labelled with either Cy3 (clones 1.14 and 5B5) or Cy5 (clones 3B12 and 2.8) dyes and a pooled internal standard was labelled with Cy2 dye to aid image matching and cross-gel statistical analysis [19]. Reverse labelling was also carried out on each sample to reduce dye bias. 2D-DIGE was then carried out according to manufacturer's instructions (GE Healthcare) and as previously described [7]. Gels were scanned with a Typhoon 9400 variable mode imager (GE Healthcare) and the subsequent Cy2, Cy3 and Cy5 gel images were analysed using the Differential In-gel Analysis (DIA) and Biological Variation Analysis (BVA) modules of DeCyder 6.5 software. Each experiment in the BVA module of Decyder 6.5 consisted of 36 gels including the Cy2-labelled images. Proteins were defined as differentially expressed if the observed average ratio was greater than 1.2, p < 0.01. This relatively small fold change was chosen as previously it has been shown that small increases in the levels of ER luminal chaperone proteins can have a cumulative effect on the rate of protein folding and assembly, with an associated increase in productivity of MAB in GS-NS0 cells [10].

### Protein identification by mass spectrometry

LC-MS/MS was performed on an Ultimate 3000 nanoLC system (Dionex), interfaced to an LTQ Orbitrap XL (Thermo Fisher Scientific). 2D gel spots were excised from Coomassie-stained preparative gels containing 400 μg of protein per gel, then destained with a solution containing 100 mM ammonium bicarbonate/acetonitrile (ACN) (1:1, vol/vol) and incubated with occasional vortexing for 30 min. Samples were then dehydrated by the addition of neat ACN and swelled by rehydration in a digestion buffer containing 12.5 ng/μL of trypsin (Promega, sequencing grade) in 10 mM ammonium bicarbonate containing 10% (vol/vol) ACN at 37°C overnight. Peptides were extracted in extraction buffer (1:2 (vol/vol) 5% formic acid/ACN), incubated for 15 min at 37°C on a shaker and dried down in a vacuum centrifuge. Tryptic peptides were re-dissolved in 10 μL of 0.1% formic acid containing 2% ACN. 5 μL of sample was loaded onto a trapping column packed with C18, PepMAP100 (Dionex), at a flow rate of 20 μL/min in 0.1% formic acid. After 5 minutes of washing, peptides were eluted into a C18 PepMAP100 nanocolumn (15 cm × 75 μm ID, 3 μm particles) (Dionex) at a flow rate of 350 nL/min. Peptides were separated using the mobile phase gradient: from 5 to 50% of solvent B in 30 min, and from 50 to 90% B in 5 min. Solvent A was 98:2 H_2_O:ACN (v/v) containing 0.1% formic acid; solvent B was 2:98 H_2_O:ACN (v/v) containing 0.1% formic acid. LC-MS/MS data was acquired in data-dependent acquisition (DDA) mode controlled by Xcalibur 2.0.7 software (Thermo Fisher Scientific). A typical DDA cycle consisted of an MS scan within *m*/*z *300-2000 performed under the target mass resolution of 60,000 (full width at half maximum) followed by MS/MS fragmentation of the six most intense precursor ions under normalised collision energy of 35% in the linear trap. Database searches were performed using TurboSEQUEST software (Bioworks Browser v3.3.1) (Thermo Fisher Scientific) using the UniProtKB/SwissProt database (Swissprot fasta database was downloaded on 26/07/2010 at 21:41 from ftp://ftp.ncbi.nlm.nih.gov/blast/db/FASTA). The following filters were applied: for charge state 1, X_Corr _> 2.0; for charge state 2, X_Corr _> 2.2; for charge state 3, X_Corr _> 2.5. Artificial modifications of peptides (carbamidomethylation of cysteines and partial oxidation of methionines) were considered. Searches were also carried out allowing for one missed cleavage. Protein identifications were accepted if they had at least 2 matched identified peptides and passed relevant statistical criteria.

### Western blotting

10 μg of protein samples were prepared in SDS-PAGE sample buffer (Sigma), heated at 100°C for 5 min and cooled on ice prior to loading onto 12% NuPAGE Bis-Tris gels (Invitrogen). Electrophoretic transfer, blocking and development of western blots were carried out as described previously [7]. Blots were probed with 1/5000 dilution of rabbit anti-human HSPD1 antibody (Abcam, ab46798) in Tris-buffered saline containing 0.1% Tween 20 (TBST) and 1/5000 dilution in TBST of mouse anti-rabbit GAPDH monoclonal antibody (Abcam ab8245) (internal loading control).

## Results

### Analysis of sustained MAB productivity over 10 days of culture

**Figure 1:**
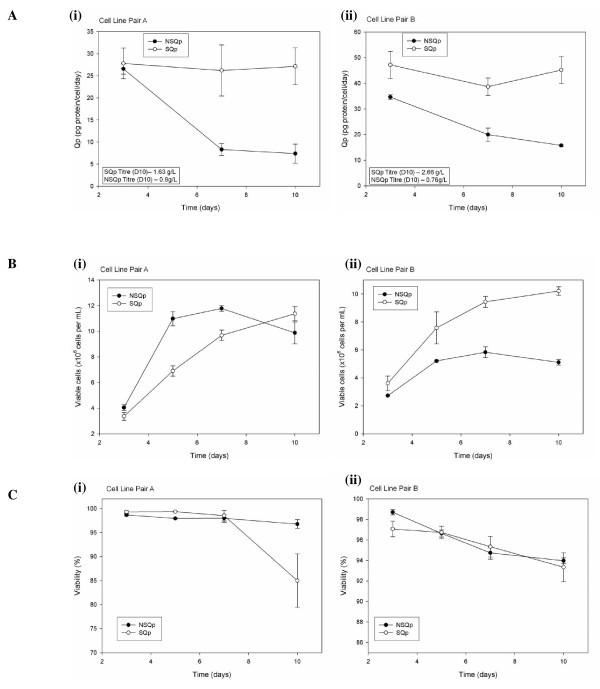
**Qp measurements for each of cell line pairs A and B with associated viable cell densities**. **A**. Qp measurements over time in culture for cell line pairs A and B. Qp measurements taken at days 3, 7 and 10. **(i) **Cell Line Pair A; **(ii) **- Cell Line Pair B. Final product titres measured at Day 10 (D10) are also included with the graphs. **B**. Growth and **C**. % viability of each culture for cell line pairs A and B. Viable cell densities were taken at days 3, 5, 7 and 10; **(i) **- Cell Line Pair A; **(ii) **- Cell Line Pair B.

Four CHO MAB-secreting cell lines were used in the study; two lines which were able to sustain productivity up to 10 days of culture were paired with two lines which were unable to sustain productivity over 10 days in culture. The cell lines which were able to sustain high productivity over 10 days in culture were labelled 'Sustained Qp (SQp)' and those that could not sustain high productivity were labelled 'Non-sustained Qp (NSQp)'. The paired comparisons were designated Cell Line Pair A (clones 3B12 (SQp) versus 5B5 (NSQp)) and Cell Line Pair B (clones 2.8 (SQp) versus 1.14 (NSQp)). The cell lines selected were paired as they were in an effort to minimize variables outside of the observed phenotype, as much as possible (see Materials and Methods above). Qp was measured for each cell line sample at Days 3, 7 and 10 (Figure 1A), and associated viable cell densities (VCDs) for each sample is shown in Figures 1B and 1C. In the two SQp cell lines, Qp remained high for the duration of the 10 day culture process. However in the case of the two NSQp cell lines, Qp fell dramatically by Day 7 and this decrease in Qp did not recover at Day 10. Therefore, these Qp measurements clearly show a difference between the cell line pairs A and B. The final product titres were also measured and calculated on Day 10 at the end of the process; for Cell Line Pair A these values averaged at 1.63 g/L for the SQp and 0.9 g/L for the NSQp cell lines; for Cell Line Pair B, product titres averaged at 2.66 g/L for the SQp and 0.76 g/L for the NSQp cell lines. From this data, the value of the sustained Qp phenotype is clear, i.e. in Cell Line Pair A where the initial Qp for both cell lines was similar and both cell lines grew to comparable VCDs, the SQp cell line generated 1.8-fold more protein over the duration of the culture through maintaining its Qp.

### Proteomic analysis of Cell Line Pairs A and B

**Figure 2:**
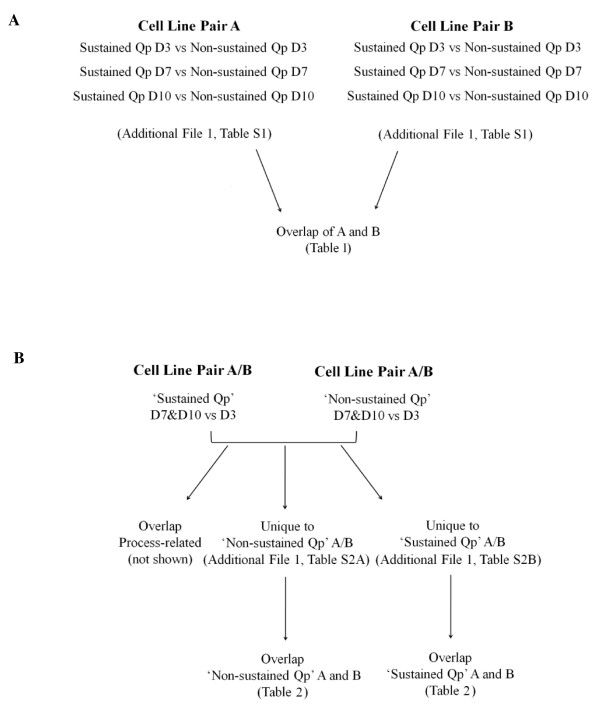
**Experimental design for proteomic analysis**. Outline of the two approaches (A and B) used to generate lists of proteins that are differentially expressed that may be associated with sustaining high productivity over a 10 day bioprocess culture.

Two approaches were used to generate lists of differentially expressed proteins. In the first approach, as outlined in Figure 2A, individual time point samples were compared between the 'SQp' and 'NSQp' samples for each pair of cell lines (i.e. SQp Day 3 v NSQp Day 3, and similarly for Days 7 and 10). The second approach involved a time-course proteomic analysis, as outlined in Figure 2B, where 'SQp' or 'NSQp' samples from Day 7 and Day 10 were grouped together and compared with Day 3 samples for each pair of cell lines in order to look for protein expression changes that possibly tracked the SQp phenotype, i.e. showing altered expression at later stages of culture compared to Day 3. Both these approaches were used to gain an understanding of the inherent differences between the SQp and NSQp cell lines (Approach 1), and further see if there are protein changes in either the SQp or the NSQp cell lines that possibly 'track' the sustained productivity phenotype through a time course analysis of the samples (Approach 2).

For image analysis of the 2D-DIGE gels, the Differential In-gel Analysis (DIA) module of Decyder was first used to merge the Cy2, Cy3 and Cy5 images for each individual gel and to detect spot boundaries in order to calculate normalised spot volumes (protein abundance). The Biological Variation Analysis (BVA) module of Decyder was then used to match spot patterns across all gels for comparative cross-gel statistical analysis with protein abundance differences normalised against the Cy2 intensity for any given spot. Operator intervention was required at this stage for removal of spots arising from dust particles and other non-protein material. Landmarks were also manually defined in the gel to increase the accuracy of the spot matching algorithm. Comparison of normalised Cy3 and Cy5 spot volumes with the corresponding Cy2 standard spot volumes within each gel gave a standardised abundance. Each of the two experiments (i.e. Cell Line Pair A and Cell Line Pair B) in the BVA module of Decyder 6.5 consisted of 36 gels (18 for the NSQp cell line and 18 for the SQp cell line); this included three biological replicate gels plus a reverse labelled technical replicate gel for each cell line sample at the three time points. The number of detected spots on each gel averaged around 2000 per gel. After spot matching and filtering, 824 proteins were found to be present in all 36 gels for Cell Line Pair A and 1028 proteins present in all 36 gels for Cell Line Pair B. These protein spots were then used for subsequent comparative experiments within each cell line pair.

#### Approach 1

**Figure 3:**
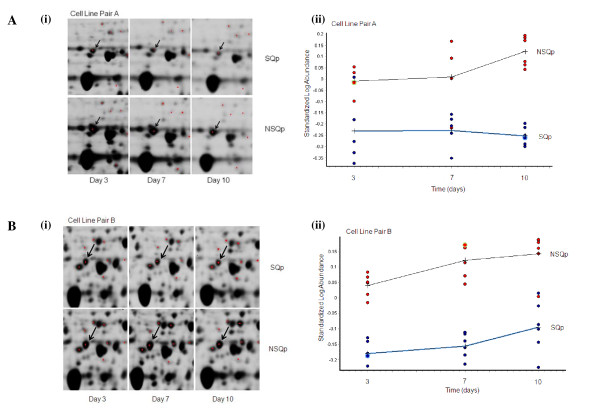
**Altered expression of Glucose 6 phosphate-1 dehydrogenase (G6PD)**. Graphical representation and zoomed in regions of 2D-DIGE gel images to demonstrate altered expression of one of the differentially expressed proteins common to cell line pairs A and B, i.e. Glucose 6 phosphate-1 dehydrogenase (G6PD), decreased in both 'SQp' cell lines compared to 'NSQp' cell lines at all time points over 10 days of culture. G6PD expression in **(A) **Cell Line Pair A and **(B) **Cell Line Pair B. **(i) **Left panel, zoomed in regions of 2D-DIGE gel images of G6PD at days 3, 7 and 10; **(ii) **Right panel, graphical representation from DeCyder 6.5 software analysis of the standardized log abundance of the spot intensities of G6PD at days 3, 7 and 10. (Blue data points - 'SQp' samples; red data points - 'NSQp' samples).

In the first approach, as outlined in Figure 2A, proteomic analyses of cell line pair A at each time point resulted in the identification of 58 differentially expressed proteins which are outlined in Additional File 1, Table S1. Similar analysis of cell line pair B resulted in the identification of 50 differentially expressed proteins and are also outlined in Additional File 1, Table S1. The proteins identified in these two lists from Cell Line Pairs A and B were overlapped to find common proteins differentially expressed between the paired comparisons, and these are outlined in Table 1 with the associated direction of expression (either increased or decreased). From Table 1, 16 proteins were found to be common between the two lists with 11 differentially expressed in the same direction, AKR1B8, ANXA1, ANXA4, EIF3I, G6PD, HSPD1, HSPA8, HSP90B1, NUDC, PGAM-1, and RUVBL1. Five proteins were differentially expressed in opposite directions, ALDH2, EEF1D, RUVBL2, PAK2 and TPI. An example of the 2D-DIGE images from one of these proteins found to be differentially expressed in both comparators, Glucose 6 phosphate-1 dehydrogenase, (i.e. decreased at all time points in the SQp cell lines compared to the NSQp cell lines) is shown in Figure 3.

**Table 1:** List of differentially expressed proteins that are common between cell line pairs A and B by comparing all 'Non-sustained Qp (NSQp)' samples to 'Sustained Qp (SQp)' samples at days 3, 7 and 10.

			Cell Line Pair A	Cell Line Pair B
**Accession no**.	Gene Name	Protein Name	Direction of expression (SQp v NQp)	Direction of expression (SQp v NQp)
***Proteins differentially regulated in the same direction***				
O08782	AKR1B8	Aldose reductase-related protein 2	UP	UP
P07150	ANXA1	Annexin A1	DOWN	DOWN
P97429	ANXA4	Annexin A4	DOWN	DOWN
Q13347	EIF3I	Eukaryotic translation initiation factor 3 subunit 1	UP	UP
O55044	G6PD	Glucose-6-phosphate 1-dehydrogenase	DOWN	DOWN
P19378	HSPA8	Heat shock cognate 71 kDa protein	UP	UP
P14625	HSP90B1	Endoplasmin	UP, DOWN (2 spots on gel)	UP
P18687	HSPD1	60 kDa heat shock protein, mitochondrial	UP	UP
Q63525	NUDC	Nuclear migration protein nudC	DOWN	DOWN (Day 7 only)
Q9DBJ1	PGAM1	Phosphoglycerate mutase 1	UP	UP
Q9Y265	RUVBL1	RuvB-like 1	UP	UP
***Proteins differentially regulated in the opposite direction***				
P47738	ALDH2	Aldehyde dehydrogenase, mitochondrial	UP	DOWN
P29692	EEF1D	Elongation factor 1-delta	UP	DOWN
Q9Y230	RUVBL2	RuvB-like 2	UP	DOWN
Q64303	PAK2	Serine/threonine-protein kinase PAK 2	DOWN	UP (Day 10 only)
P48500	TPI1	Triosephosphate isomerase	DOWN	UP

Samples from all 3 time points were also combined into one analysis and compared. DOWN - decreased in the SQp cells compared to NSQp cells; UP - increased in SQp cells compared to NSQp cells. Accession number is from UniProtKB/Swiss-Prot protein database. The full protein lists from each cell line pair are in Additional File 1, Table S1.

#### Approach 2

A time-course proteomic analysis was also carried out, as outlined in Figure 2B, where 'SQp' or 'NQp' samples from Day 7 and Day 10 were grouped together and compared with Day 3 samples for each pair of cell lines. The purpose of this analysis was to identify those proteins whose expression changes over the course of the production fed batch for each of the cell lines, and then separate those that are unique to the 'SQp' or 'NSQp' lines from those that are common to all cell lines and therefore are what we are calling 'process-related'. The 'SQp' and 'NSQp' lists from cell line pair A were overlapped to look for uniquely differentially expressed proteins. Proteins showing 'process-related' changes (i.e. proteins whose expression levels change as a result of culture conditions over time) in both 'SQp' and 'NSQp' cell lines were removed from the analysis. 14 proteins were identified as changed only in the 'NSQp' cell line, and 14 proteins identified as changed only in the 'SQp' cell line (see Additional File 1, Tables 2A and 2B) for cell line pair A. The same approach was used for cell line pair B, with 10 proteins identified as changed only in the 'NSQp' cell line and 8 identified as changed only in the 'SQp' cell line (Additional File 1, Tables 2A and 2B). These two 'unique' lists from both cell line pairs were subsequently overlapped to identify a second list of proteins that may be linked with the ability of a cell line to sustain productivity over a 10 day bioprocess culture, and are outlined in Table 2. Overlapping the 'NSQp' lists between the two cell line pairs A and B revealed that two proteins, HSPD1 and EIF3I, were common to both comparators and were differentially expressed in the same direction in both cases (i.e. both decreased at days 7 and 10 compared to day 3). Similarly, the 'SQp' lists from both comparators were overlapped and two proteins, CNN3 and AKRIB8, were common to both lists and were both decreased at day 7 and 10 compared to day 3. Three of these proteins (HSPD1, EIF3I and AKRIB8) were also found to overlap with Table 1, reconfirming these proteins as potentially playing a role in sustaining recombinant protein production in CHO cell lines.

**Table 2:** List of unique differentially expressed proteins that are common between cell line pairs A and B by comparing 'Non-sustained Qp' or 'Sustained Qp' samples at Days 7&10 with Day 3 samples.

**Accession no**.	Gene Name	Protein name	Cell Line Pair A **(Direction of expression**, D7&10 v D3)	Cell Line Pair B **(Direction of expression**, D7&10 v D3)
***Unique to NSQp***				
P18687	HSPD1	60 kDa heat shock protein, mitochondrial	Down	Down
Q13347	EIF3I	Eukaryotic translation initiation factor 3 subunit 1	Down	Down
***Unique to SQp***				
P37397	CNN3	Calponin-3	Down	Down
O08782	AKR1B8	Aldose reductase-related protein 2	Down	Down

DOWN - decreased in Day 7&10 samples compared to Day 3 samples; UP - increased in Day 7&10 samples compared to Day 3 samples. Accession number is from UniProtKB/Swiss-Prot protein database. The full protein lists from each cell line pair are in Additional File 1, Table S2 (A and B).

**Figure 4:**
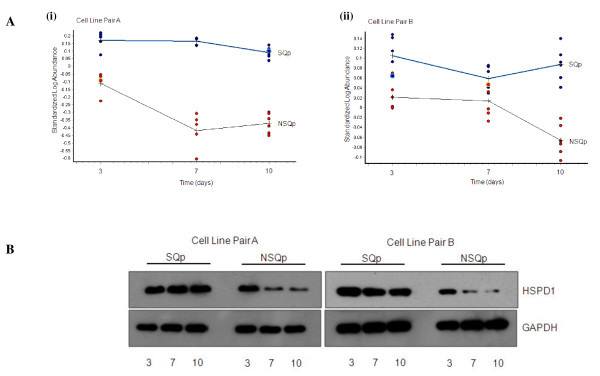
**Expression of HSPD1 'tracking' the sustained productivity phenotype: 2D-DIGE and western blot analysis to demonstrate HSPD1 protein expression 'tracking' the sustained productivity phenotype in the SQp and NSQp cell lines from Cell Line Pairs A and B**. **A**. Graphical representation of 2D-DIGE gel protein spots showing the standardized log abundance of spot intensities at days 3, 7 and 10 from DeCyder analysis of **(i) **Cell Line Pair A and **(ii) **Cell Line Pair B. Blue data points - 'SQp' samples; red data points - 'NSQp' samples. **B**. Western blot analysis of HSPD1 protein expression in the two cell line pairs, A and B. GAPDH was used to demonstrate equal loading between samples.

Figure 4 shows an example of one of these proteins, HSPD1, whose expression levels appear to follow a pattern of protein expression that mirrors the altered Qp of the cell line pairs over the 10 day culture process. In the case of the 'SQp' cell lines, HSPD1 protein expression remains constant over the three time points while the levels appear to decrease at days 7 and 10 for the 'NSQp' cell lines (Figure 4A) similar to the fall off in Qp at days 7 and 10 for these cell line samples (Figure 1A). This was reconfirmed through western blot validation using an antibody against HSPD1 (Figure 4B).

#### Overall summary of proteomic datasets

Overall, proteomic analysis of the two cell line pairs has identified 154 proteins that are possibly related to a sustained productivity phenotype; (i.e. 58 for Cell Line Pair A and 50 for Cell Line Pair B in Additional File 1, Table S1, 24 proteins from Cell Line Pairs A and B in Additional File 1, Table S2A, and 22 proteins from Cell Line Pairs A and B in Additional File 1, Table S2B). These 154 proteins represent 89 distinct proteins due to overlap between the different lists and also due to the fact that a number of proteins, such as ALDH2, ANXA1, HSPA8, HSP90B, TCP1, VCP, RPSA, SERPINB1A, UBEK2, were identified more than once on the 2D gels which could be indicative of posttranslational modifications. For example, HSPA8 and VCP are both known to be potentially phosphorylated [20,21]. A number of the proteins from this list of 89 proteins (i.e. RPLP0, ENO1, CNN3, ANXA5, EEF1D, LGALS1, HSPA8, HSPB1, HMGCS1, NSFL1C, RPIA and TPT1) were found to overlap with a previous study from our laboratory related to proteomic profiling of low and high productivity phenotypes from recombinant CHO cells [7].

When combining the overlap lists (Tables 1 and 2) from the two proteomic analysis approaches used to yield a list of proteins potentially related to a sustained productivity phenotype, twelve of these 89 proteins were found to be differentially expressed in the same direction, i.e. AKRIB8, ANXA1, ANXA4, EIF3I, G6PD, HSPA8, HSP90B1, HSPD1, NUDC, PGAM1, RUVBL1 and CNN3.

## Discussion

Cell specific productivity is a function of a multitude of cellular processes including gene transcription, mRNA stability, translation, glycosylation, polypeptide folding, ER-associated degradation and inter-vesicular transport of fully assembled proteins [5]. Few studies have investigated the molecular basis of how recombinant cell lines are capable of sustaining productivity of a product of interest over the duration of a culture process. From this study, we have identified 89 distinct proteins that are differentially expressed in two pairs of cell lines that substantially differ in their ability to sustain Qp over the full duration of an optimized, 10-day fed-batch culture. Overlapping the proteomic lists generated from analysis of the two cell line pairs revealed a list of 12 proteins, common between the two sets of analyses that were differentially regulated in the same direction (Tables 1 and 2). Some of these 12 proteins, particularly HSPD1 and EIF3I, were found to 'track' the sustained Qp phenotype, i.e. HSPD1 and EIF3I protein expression remained constant in the SQp cell lines, in contrast to the NSQp cell lines

From the 12 differentially expressed proteins, four of the proteins that show increased expression in the SQp cell lines are known to be involved in protein translation (EIF3I) [22] and protein folding (HSPD1, HSPA8 and HSP90) [23-25], suggesting that the SQp cell lines have possibly a greater efficiency in sustaining protein synthesis and folding of polypeptides over the duration of the 10 day bioprocess culture. HSPD1, HSPA8 and HSP90 have all been previously been shown in other studies to have increased expression in higher producing cell lines [9,10]. HSPD1 (HSP60) is a member of the chaperonin family and is essential for the folding and assembly of newly imported proteins in the mitochondria [24]. Increased expression of HSPD1 has been found to be associated with increased MAB production in GS-NS0 cells [10]. HSPA8 is a member of the HSP70 protein family which is known to have anti-apoptotic properties [26]. As a result, HSPA8 has also been used as an engineering target to prolong culture viability resulting in increased product titre in a number of cell lines including CHO, NS0 and BHK-21 [27-29]. HSP90B1 (also known as GRP94 or endoplasmin) is a highly conserved ER luminal molecular chaperone that has key roles in folding newly synthesized proteins or stabilizing and refolding denatured proteins after stress. HSP90 proteins normally interact with other co-chaperones as part of a multi-protein complex (with BiP) associated with nascent Ig heavy chain synthesis in the ER [23,30]. Previous proteomic studies have shown that HSP90 exhibits an increase in abundance that correlates with increased qMAB in NS0 cells [9,10].

The list of 12 overlapping proteins also included three proteins involved in glucose metabolism, with PGAM1 and AKRIB8 showing increased expression while G6PD showed decreased expression in both cell line pairs. G6PD (glucose 6 phosphate dehydrogenase) is involved in the production of NADPH, a key electron donor in the defense against oxidizing agents and in reductive biosynthetic reactions. PGAM1 is a glycolytic enzyme that catalyses the interconversion of 3-phosphoglycerate and 2-phosphoglycerate. AKRIB8 (aldo-keto reductase) is a phase I metabolising enzyme that catalyses the reduced nicotinamide adenine dinucleotide (phosphate) (NAD(P)H)-dependent reduction of carbonyl groups to yield primary and secondary alcohols on a wide range of substrates. It has been previously suggested based on proteomic analysis of high MAB productivity in NS0 cells, that up-regulation of processes such as energy metabolism may be involved in the improved production of MAB [31]. During B cell differentiation, B cells may anticipate their secretory role in a multistep process; firstly by up-regulating the expression of proteins involved in metabolism followed by an expansion of the secretory machinery to accommodate the mass production of antibodies [32]. It has also been previously suggested that the complex trait of hyperproductivity in recombinant mammalian cell lines could have some similarities to the differentiation of non-secretory B cells to plasma cells [33] with higher producing cells requiring higher energy levels. Perhaps some parallels could be drawn to our study in that the cells that are able to sustain productivity have an increased metabolic capacity which enables them to sustain productivity of MAB over the duration of the 10 day bioprocess culture.

Other proteins with a variety of cellular functions present on the overlap list include RUVBL1, ANXA1, ANXA4, NUDC and CNN3. RUVBL1 is an ATP-binding protein that belongs to the AAA+ family of ATPases, which have been implicated in a variety of cellular processes such as transcription, small ribonucleolar protein (snoRNPs) complex assembly, cellular transformation, metastasis and apoptosis [34]. ANXA1 and ANXA4 are both members of the annexin family of Ca(2+)-dependent phospholipid binding proteins. ANXA1 is involved in the regulation of cell proliferation and apoptosis [35,36] while ANXA4 can promote membrane fusion and is involved in exocytosis [37]. NUDC (nuclear distribution gene homologue) plays a role in cell division through the regulation of cytoplasmic dynein [38]. Through its interaction with dynein NUDC is also involved in the perinuclear localization of the Golgi apparatus and lysosomes and endocytic vesicles transport [39]. CNN3 (calponin-3) is an actin-associated protein and is involved in cellular architecture and cell motility [40]. Further work on these proteins is required to understand the role they play in the sustained Qp phenotype.

## Conclusions

We have described here the first attempt to unravel some of the molecular basis for a sustained high Qp phenotype in CHO cells. These proteins may be useful as biomarkers to pre-select cell line clones at an earlier stage of cell line development to enrich for clones with a stable Qp phenotype. Moreover, it is possible that many of these proteins could be successfully manipulated or engineered so as to elicit the very phenotype we have characterized. Indeed, individual target engineering may only be the first step into pathway engineering, as this phenotype is likely to represent a complex interplay between metabolic and signalling pathways. Nevertheless, the utility of biomarkers is that they provide a powerful tool for screening a diverse population of clones in order to identify those with predictably preferred characteristics. Future research into both of these investigational lines will be of great value to bioprocess science.

## List of Abbreviations

CHO: Chinese Hamster ovary; MAB: Monoclonal antibody; VCD: viable cell density; SQp: sustained productivity; NSQp: non-sustained productivity; 2D-DIGE: 2D differential gel electrophoresis; LC-MS: Liquid chromatography-mass spectrometry.

## Competing interests

The authors declare that they have no competing interests. MM, PG and ML are employees of Pfizer.

## Authors' contributions

PM and MM designed the experimental approach. PM and JK carried out the 2D DIGE analysis. PM and MH did the mass spectrometry identification. PM, PD, NB, FOS, PG, CC, MM were involved with result interpretation and manuscript preparation. ML and MC conceived the study and participated in the study design and coordination. All authors read and approved the final manuscript.

## Supplementary Material

Additional File 1 **Table S1: List of differentially expressed proteins identified by 2D-DIGE and LC-MS/MS from cell line pairs A and B by comparing 'Non-sustained Qp' samples to 'Sustained Qp' samples at each time point**. Ratios outlined in italics are for information only to show the general trend of protein expression, as they have not passed the statistical criteria outlined for DeCyder analysis of the 2D-DIGE images, i.e. average ratio greater than 1.2, p < 0.01. Mass Spectrometry protein identifications were accepted if they had at least 2 matched identified peptides and passed relevant statistical criteria including XCorr Scores (i.e. for charge state 1, X_Corr _> 2.0; for charge state 2, X_Corr _> 2.2; for charge state 3, X_Corr _> 2.5). **Table S2**: **A**. List of unique differentially expressed proteins identified by 2D-DIGE and LC-MS/MS from cell line pairs A and B by comparing 'Non-sustained Qp' cell lines at Days 7&10 with Day 3 samples. **B**. List of unique differentially expressed proteins identified from cell line pairs A and B by comparing 'Sustained Qp' cell lines at Days 7&10 with Day 3 samples. Mass Spectrometry protein identifications were accepted if they had at least 2 matched identified peptides and passed relevant statistical criteria including XCorr Scores (i.e. for charge state 1, X_Corr _> 2.0; for charge state 2, X_Corr _> 2.2; for charge state 3, X_Corr _> 2.5). Click here for file
