# Supplementary material for: Sustained productivity in recombinant Chinese Hamster Ovary (CHO) cell lines: proteome analysis of the molecular basis for a process-related phenotype
Source: BMC Biotechnol. 2011 Jul 24;11:78. doi: 10.1186/1472-6750-11-78 (PMC3170212; doi:10.1186/1472-6750-11-78)
Supplement: Additional File 1 — Table S1: List of differentially expressed proteins identified by 2D-DIGE and LC-MS/MS from cell line pairs A and B by comparing 'Non-sustained Qp' samples to 'Sustained Qp' samples at each time point. Ratios outlined in italics are for information only to show the general trend of protein expression, as they have not passed the statistical criteria outlined for DeCyder analysis of the 2D-DIGE images, i.e. average ratio greater than 1.2, p < 0.01. Mass Spectrometry protein identifications were accepted if they had at least 2 matched identified peptides and passed relevant statistical criteria including XCorr Scores (i.e. for charge state 1, XCorr > 2.0; for charge state 2, XCorr > 2.2; for charge state 3, XCorr > 2.5). Table S2: A. List of unique differentially expressed proteins identified by 2D-DIGE and LC-MS/MS from cell line pairs A and B by comparing 'Non-sustained Qp' cell lines at Days 7&10 with Day 3 samples. B. List of unique differentially expressed proteins identified from cell line pairs A and B by comparing 'Sustained Qp' cell lines at Days 7&10 with Day 3 samples. Mass Spectrometry protein identifications were accepted if they had at least 2 matched identified peptides and passed relevant statistical criteria including XCorr Scores (i.e. for charge state 1, XCorr > 2.0; for charge state 2, XCorr > 2.2; for charge state 3, XCorr > 2.5). [file 1472-6750-11-78-S1.PDF]

Additional File 1, Table S1

Accession number	Gene Name	Protein Name	Ratio SQp D3 v NSQp D3	Ratio SQp D7 v NSQp D7	Ratio SQp D10 v NSQp D10
Cell Line Pair A					
P63324	RPS12	40S ribosomal protein S12	1.39	2.15	2.27
P14869	RPLP0	60S acidic ribosomal protein P0	14.31	7.5	4.56
P18687	HSPD1	60 kDa heat shock protein, mitochondrial	1.8	3.73	2.87
P47738	ALDH2	Aldehyde dehydrogenase, mitochondrial	1.42	1.53	1.73
P4773	ALDH2	Aldehyde dehydrogenase, mitochondrial	2.12	2.06	1.94
O08782	AKR1B8	Aldose reductase-related protein 2	1.82	1.7	*1.2*
P07150	ANXA1	Annexin A1	-2.67	-1.37	-3.32
P04083	ANXA1	Annexin A1	-9.79	-9.49	-12.34
P55260	ANXA4	Annexin A4	-1.32	-1.42	-1.67
P00173	CYB5A	Cytochrome b5	-5.91	-5.21	-2.81
P29692	EEF1D	Elongation factor 1-delta	2.23	2.1	1.92
Q9D8N	EEF1G	Elongation factor 1-gamma	2.34	2.22	2.2
Q13347	EIF3I	Eukaryotic translation initiation factor 3 subunit I	1.44	1.93	1.35
O55044	G6PD	Glucose-6-phosphate 1-dehydrogenase	-1.79	-1.78	-2.38
P11352	GPX1	Glutathione peroxidase 1	1.32	2	2.29
P19378	HSPA8	Heat shock cognate 71 kDa protein	1.5	1.94	1.84
P19378	HSPA8	Heat shock cognate 71 kDa protein	1.42	1.69	1.68
P14625	HSP90B1	Endoplasmin	3.69	2.19	1.63
P14625	HSP90B1	Endoplasmin	-1.42	-1.51	-10.55
Q99729	HNRNPAB	Heterogeneous nuclear ribonucleoprotein A/B	5.14	5.1	2.46
Q60432	HYOU1	Hypoxia up-regulated protein 1	4.66	3.79	2.46
P12269	IMPDH2	Inosine-5'-monophosphate dehydrogenase 2	1.94	*-1.15*	-1.93
Q9D6R2	IDH3A	Isocitrate dehydrogenase [NAD] subunit alpha, mitochondrial	1.52	2.05	1.94
Q13907	IDI1	Isopentenyl-diphosphate Delta-isomerase 1	-5.24	-2.43	-1.97
Q9UIC8	LCMT1	Leucine carboxyl methyltransferase 1	3.5	1.75	1.54
P70699	GAA	Lysosomal alpha-glucosidase	5.41	6.62	5.79
Q9EQK5	MVP	Major vault protein	2.04	2.36	3.18
Q9D880	TIMM50	Mitochondrial import inner membrane translocase subunit TIM50	-3.77	-2.12	-1.77
O70378	COX4NB	Neighbor of COX4	1.8	2.64	2.31
Q63525	NUDC	Nuclear migration protein nudC	-9.26	-7.83	-7.45
Q8K3U7	PRDX2	Peroxiredoxin-2	1.56	2.05	2.33

Q9DBJ1	PGAM1	Phosphoglycerate mutase 1	1.66	4.85	4.23
P61759	VBP1	Prefoldin subunit 3	12.26	6.51	2.25
P67779	PHB	Prohibitin	-2.28	-1.52	-1.64
P60901	PSMA6	Proteasome subunit alpha type-6	2.16	4.38	3.53
P70195	PSMB7	Proteasome subunit beta type 7	1.78	2.03	1.45
Q9R1P1	PSMB3	Proteasome subunit beta type-3	*-1.16*	*-1.1*	-2.86
P05964	S100A6	Protein S100-A6	-1.6	*-1.08*	1.87
P26043	RDX1	Radixin	1.77	1.31	*-1.04*
P34022	RANBP1	Ran-specific GTPase-activating protein	2.99	5.18	7.6
Q9Y265	RUVBL1	RuvB-like 1	2.2	3.56	2.83
Q9Y230	RUVBL2	RuvB-like 2	3.31	2.52	2.82
Q64303	PAK2	Serine/threonine-protein kinase PAK 2	-1.2	-1.83	-2.2
Q9NR45	NANS	Sialic acid synthase	-3.08	-2.02	-1.94
O54981	STIP1	Stress-induced-phosphoprotein 1	1.65	2.11	1.73
P11984	TCP1	T-complex protein 1 subunit alpha	3.64	3.81	3.08
P11984	TCP1	T-complex protein 1 subunit alpha	-2.94	-1.7	-2.01
P50990	CCT8	T-complex protein 1 subunit theta	3.37	5.04	2.91
Q8VI73	TALDO1	Transaldolase	15.04	13.19	9.49
P46462	VCP	Transitional endoplasmic reticulum ATPase (Valosin-containing protein)	-2.87	-4.72	-4.23
P46462	VCP	Transitional endoplasmic reticulum ATPase (Valosin-containing protein)	-4.78	-8.77	-11.12
P13693	TPT1	Translationally-controlled tumor protein	1.55	2.53	2.24
P48500	TPI1	Triosephosphate isomerase	-1.88	-1.54	-1.69
Q9BQE3	TUBA1C	Tubulin alpha-1C chain	*1.04*	-1.36	-1.84
P68371	TUBB2C	Tubulin beta-2C chain	*1.24*	1.44	2.5
P69893	TUBB5	Tubulin beta-5 chain	6.7	6.63	4.58
P31254	UBEIAY	Ubiquitin-like modifier-activating enzyme 1 Y	-5.83	-5.33	-5.89
P62815	ATP6V1B2	V-type proton ATPase subunit B, brain isoform	*-1.19*	-1.59	-3.45
Cell Line Pair B					
38982	RPSA	40S ribosomal protein SA	1.25	*1.18*	1.49
P38982	RPSA	40S ribosomal protein SA	*1.16*	*1.12*	1.45
P18687	HSPD1	60 kDa heat shock protein, mitochondrial	1.21	*1.11*	1.42
P55262	ADK	Adenosine kinase (AK)	1.25	*1.15*	1.41
Q9WTP6	AK2	Adenylate kinase 2, mitochondrial	1.32	1.28	1.38
P47738	ALDH2	Aldehyde dehydrogenase, mitochondrial	-1.23	-1.25	*-1.06*
O08782	AKR1B8	Aldose reductase-related protein 2	1.85	1.5	1.43
P06733	ENO1	Alpha-enolase	-1.25	-1.21	1.2
P07150	ANXA1	Annexin A1	-1.48	-1.43	-1.49

P97429	ANXA4	Annexin A4	-1.47	-1.48	-1.43
P48036	ANXA5	Annexin A5	1.29	1.28	1.34
P37397	CNN3	Calponin-3	-1.49	-2.38	-3.01
P23528	CFL1	Cofilin-1	-1.88	-2.72	-5.86
Q68FS4	LAP3	Cytosol aminopeptidase	1.3	1.26	1.27
O08651	PHGDH	D-3-phosphoglycerate dehydrogenase	-1.24	-1.25	*-1.02*
Q62651	ECH1	Delta(3,5)-Delta(2,4)-dienoyl-CoA isomerase, mitochondrial	1.53	2.49	2.42
Q80UW8	POLR2E	DNA-directed RNA polymerases I, II, and III subunit RPABC1	-1.87	-1.66	-1.63
P29692	EEF1D	Elongation factor 1-delta	*-1.17*	-1.23	-1.29
P09445	EEF2	Elongation factor 2	*-1.06*	1.65	1.91
Q9CZR8	TSFM	Elongation factor Ts, mitochondrial	1.22	1.16	1.4
Q13347	EIF3I	Eukaryotic translation initiation factor 3 subunit I	1.2	1.22	1.53
Q9WUK2	EIF4H	Eukaryotic translation initiation factor 4H	*-1.02*	-1.35	-1.52
P48538	LGALS1	Galectin-1	-1.58	-1.24	*-1.07*
O55044	G6PD	Glucose-6-phosphate 1-dehydrogenase	-1.66	-1.89	-1.72
P48508	GCLM	Glutamate--cysteine ligase regulatory subunit	*-1.06*	-1.44	-1.58
Q60631	GRB2	Growth factor receptor-bound protein 2	*-1.03*	*-1.03*	-1.36
P19378	HSPA8	Heat shock cognate 71 kDa protein	1.45	1.33	1.5
P14625	HSP90B1	Endoplasmin	1.2	1.23	1.28
Q13907	IDI1	Isopentenyl-diphosphate Delta-isomerase 1	1.48	1.57	1.58
Q4G075	SERPINB1A	Leukocyte elastase inhibitor A	-1.5	-1.36	-1.32
Q4G075	SERPINB1A	Leukocyte elastase inhibitor A	-2.78	-1.91	-1.49
Q63525	NUDC	Nuclear migration protein nudC	*-1.09*	-1.2	*-1.14*
Q9DBJ1	PGAM1	Phosphoglycerate mutase 1	1.35	1.28	1.66
P27773	PDIA3	Protein disulfide-isomerase A3	-1.25	-1.3	-1.19
Q8R4U2	P4HB	Protein disulfide-isomerase	-1.58	-1.52	-1.36
Q64311	NTAN1	Protein N-terminal asparagine amidohydrolase	-1.21	*-1.09*	-1.26
Q8K183	PDXK	Pyridoxal kinase	1.2	1.22	1.53
Q8WUD1	RAB2B	Ras-related protein Rab-2B	-1.56	-1.66	-1.6
P47968	RPIA	Ribose-5-phosphate isomerase	1.4	1.43	1.61
Q9Y265	RUVBL1	RuvB-like 1	1.44	1.31	1.48
Q9Y230	RUVBL2	RuvB-like 2	-1.21	-1.32	-1.31
Q64303	PAK2	Serine/threonine-protein kinase PAK 2	*1.17*	*1.16*	1.28
P08228	SOD1	Superoxide dismutase [Cu-Zn]	-1.39	-1.24	-1.38
P48500	TPI1	Triosephosphate isomerase	2.19	2.19	2.35
P48500	TPI1	Triosephosphate isomerase	2.34	2.61	2.68
Q6P7B0	WARS	Tryptophanyl-tRNA synthetase, cytoplasmic	*-1.14*	*1.18*	1.38

P48428	TBCA	Tubulin-specific chaperone A	1.39	1.32	1.4
P61085	UBE2K	Ubiquitin-conjugating enzyme E2 K	2	1.8	1.84
P61085	UBE2K	Ubiquitin-conjugating enzyme E2 K	-*1.15*	-1.28	-1.22
P61085	UBE2K	Ubiquitin-conjugating enzyme E2 K	1.21	-1.4	-1.78

Additional File 1, Table S2

A –Proteins differentially expressed only in the NSQp cell lines, when comparing Day 7&10 to Day 3 samples

Accession number	Gene Name	Protein Name	Ratio D7&D10 v D3
Cell Line Pair A			
P63324	RPS12	40S ribosomal protein S12	-1.71
P18687	HSPD1	60 kDa heat shock protein, mitochondrial	-1.9
Q13347	EIF3I	Eukaryotic translation initiation factor 3 subunit I	-1.33
P14625.	HSP90B1	Endoplasmin	2.12
P42930	HSPB1	Heat shock protein beta-1	-1.48
Q9DBJ1	PGAM1	Phosphoglycerate mutase 1	-2.37
P17918	PCNA	Proliferating cell nuclear antigen (PCNA)	-1.63
P17918	PCNA	Proliferating cell nuclear antigen	-1.74
P34022	RANBP1	Ran-specific GTPase-activating protein	-1.96
Q9Y265	RUVBL1	RuvB-like 1	-1.45
Q64303	PAK2	Serine/threonine-protein kinase PAK 2	1.92
O54981	STIP1	Stress-induced-phosphoprotein 1	-1.42
P13693	TPT1	Translationally-controlled tumor protein	-1.84
Q9BQE3	TUBA1C	Tubulin alpha-1C chain	1.4
Cell Line Pair B			
P38982	RPSA	40S ribosomal protein SA	-1.61
P18687	HSPD1	60 kDa heat shock protein, mitochondrial	-1.22
P07150	ANXA1	Annexin A1	1.77
P23528	CFL1	Cofilin-1	4.91
Q13347	EIF3I	Eukaryotic translation initiation factor 3 subunit I	-1.28
O55044	G6PD	Glucose-6-phosphate 1-dehydrogenase	1.24
Q13907	IDI1	Isopentenyl-diphosphate Delta-isomerase 1	-1.28
P38652	PGM1	Phosphoglucomutase-1	2.4
Q8K183	PDXK	Pyridoxal kinase (Pyridoxine kinase)	-1.28
Q9Y230	RUVBL2	RuvB-like 2	1.28

Additional File 1, Table S2

B –Proteins differentially expressed only in the SQp cell lines, when comparing Day 7&10 to Day 3 samples

Accession number	Gene Name	Protein Name	Ratio D7&D10 v D3
Cell Line Pair A			
P14869	RPLP0	60S acidic ribosomal protein P0	-2.34
O08782	AKR1B8	Aldose reductase-related protein 2	-1.64
P37397	CNN3	Calponin-3	-4.85
Q9D1A2	CNDP2	Cytosolic non-specific dipeptidase	1.86
P11352	GPX1	Glutathione peroxidase 1	1.32
P13704	HMGCS1	Hydroxymethylglutaryl-CoA synthase, cytoplasmic	-3.6
P12269	IMPDH2	Inosine-5'-monophosphate dehydrogenase 2	-2.6
Q13907	IDI1	Isopentenyl-diphosphate Delta-isomerase 1	1.58
Q9UIC8	LCMT1	Leucine carboxyl methyltransferase 1	-2.01
Q9EQK5	MVP	Major vault protein	-1.62
O35987	NSFL1C	NSFL1 cofactor p47	-1.74
Q62422	OSTF1	Osteoclast-stimulating factor 1	2.35
P61759	VBP1	Prefoldin subunit 3	-2.24
O14744	PRMT5	Protein arginine N-methyltransferase 5	-1.67
Cell Line Pair B			
O08782	AKR1B8	Aldose reductase-related protein 2	-1.41
P37397	CNN3	Calponin-3	-1.85
Q62651	ECH1	Delta(3,5)-Delta(2,4)-dienoyl-CoA isomerase, mitochondrial	1.74
Q80UW8	POLR2E	DNA-directed RNA polymerases I, II, and III subunit RPABC1	1.47
Q9WUK2	EIF4H	Eukaryotic translation initiation factor 4H	-1.38
P48538	LGALS1	Galectin-1	1.61
P15650	ACADL	Long-chain specific acyl-CoA dehydrogenase, mitochondrial	1.41
Q6P7B0	WARS	Tryptophanyl-tRNA synthetase, cytoplasmic	1.66
